# Evidences that SARS-CoV-2 Vaccine-Induced apoplexy may not be solely due to ASIA or VITT syndrome', Commentary on Pituitary apoplexy and COVID-19 vaccination: A case report and literature review

**DOI:** 10.3389/fendo.2023.1111581

**Published:** 2023-01-25

**Authors:** Ach Taieb, Ben Abdelkrim Asma, El Euch Mounira

**Affiliations:** ^1^ Department of Endocrinology, University Hospital of Farhat Hached, Sousse, Tunisia; ^2^ University of Sousse, Faculty of Medicine of Sousse, Sousse, Tunisia; ^3^ Laboratory of Exercise Physiology and Pathophysiology, University Hospital of Farhat Hached, Sousse, Tunisia; ^4^ Department of Internal Medicine, University Hospital of Charles Nicolle, Tunis, Tunisia

**Keywords:** COVID-19, SARS-CoV-2, vaccine, apoplexy, hypophysitis, pituitary adenoma

## 1 Introduction

The present commentary aims to discuss certain diagnostic and pathophysiological aspects of pituitary apoplexy in the case reported by Aliberti et al. (2022) (1).

The report states the occurrence of pituitary apoplexy following the third dose of Moderna vaccine. It is the fourth case, to date, to report this adverse event (2–4), the second to report it in a patient with a pituitary adenoma (4), and the first of its kind to confirm the persistent presence of traces of SARS-CoV-2 infection as confirmed by histopathological examination of a pituitary biopsy.

As a complement to the first and recently published review of pilot findings addressing SARS-CoV-2 vaccine-induced pituitary diseases (5), we will briefly discuss other interesting elements concerning this entity that will be highlighted by this report.

## 2 Pituitary adenoma patients are at a higher risk of vaccine-induced apoplexy

It is interesting to mention that before the case of Aliberti et al. (1), only Piñar-Gutiérrez et al. have reported pituitary apoplexy in a patient with a pituitary adenoma (4). The incident occurred 5 days after the first dose of AstraZeneca vaccine, and the MRI showed an adenohypophysis hemorrhage in association with a 10-mm intraglandular adenoma. Pituitary adenomas in men, if not prolactin- or growth hormone (GH)-secreting, are usually nonfunctional and large (>10 mm) (6). It is not uncommon to discover them following an event-induced apoplexy, as mentioned by the authors. In cases of apoplexy linked to vaccines, pituitary bleeding can be due either to vaccine-induced thrombophilia–thrombocytopenia (VITT) syndrome or to autoimmune/autoinflammatory syndrome induced by adjuvants (ASIA) syndrome (5). The ASIA syndrome can only be confirmed by a histopathological study. However, VITT has diagnostic criteria that have been recently updated to fit the context of post-vaccine thrombosis (7).

According to these recommendations, the presence of the following criteria is required to diagnose VITT syndrome:

1. COVID-19 vaccine at 4 to 42 days prior to symptom onset
2. any venous or arterial thrombosis
3. thrombocytopenia (platelet count <150 × 10^9^/L)
4. positive Platelet factor 4 (PF4) heparin-induced thrombocytopenia (HIT) ELISA
5. elevated D-dimer (>4× upper limit of normal)

Upon reading the case of Aliberti et al. (1), several elements suggest that these two pathophysiological hypotheses are not easily applicable in this case. Firstly, several major criteria of VITT are not fulfilled: The patient had a rapid onset of the disease (on vaccination day) and a platelet count superior to 150 × 10^9^/L (170 × 10^9^/L). Secondly, the rapid onset of the disease cannot be explained by an inflammatory or an autoimmune mechanism as such processes require a longer time interval to have clinical consequences (8). In comparison, other cases of SARS-CoV-2 vaccine-induced pituitary diseases report a delay of a few days for the beginning of symptoms (3 days on average) (9).

The rapid onset of apoplexy suggests that the patient’s pituitary gland already has fragility or a pathological state that predisposes it to bleeding. Pituitary adenoma, which is due to a richly vascularized cellular hyperplasia, can be the cause of such a delicate state (6). Several authors have already mentioned the fragility of pituitary adenomas to chemical or physical stimuli, making the risk of hemorrhage a major one (10).

## 3 An alternative hypothesis to vaccine-induced thrombophilia–thrombocytopenia or autoimmune/autoinflammatory syndrome induced by adjuvants syndrome

The originality in the case presented by Aliberti et al. (1) is the existence of a histopathological proof of asymptomatic infection by SARS-CoV-2. Several authors have confirmed the involvement of this virus in endothelial damage, which seems to explain the persistence of the “long-standing COVID-19” state (11). Two articles have even proved the possibility of pituitary insufficiencies as sequelae in previously infected patients. Urhan et al. have identified a corticotropic insufficiency in nearly 10% of cases and a GH insufficiency in nearly 50% of cases in patients infected by COVID-19, without notable hypopituitarism manifestations (12). These disorders persisted for several months after the infection and even longer (up to 15 months), as mentioned by Yoshimura et al. in their analysis (13).

We can therefore assume that in patients with an adenomatous pituitary gland weakened by an asymptomatic infection, there is an increased hemorrhagic risk caused by the vaccine. In this case, the VITT syndrome or the ASIA syndrome could initiate hemorrhage and be expressed quite rapidly, even on the first day of injection, aggravated by the fragility of the pituitary gland. This is further supported by the SARS-CoV-2 nuclear protein expression next to pituitary vessels, in the presence of an evident lymphocyte infiltrate (1). Therefore, this pathophysiological hypothesis supports the rapidity of the onset of apoplexy in both cases reported by Aliberti et al. (1) and Piñar-Gutiérrez et al. (4) since the patients had, concomitantly, a pituitary macroadenoma.

## 4 Summary and conclusions

In summary, the case of Aliberti et al. (1) gives insight or helpful information for the understanding of SARS-CoV-2 post-infectious pituitary lesions by confirming the existence of persistent endothelial damage even in asymptomatic patients or after recovery. This damage to the adenomatous pituitary gland could further weaken it and cause hemorrhage upon exposure to the COVID-19 vaccine. This case emphasizes the need for clinicians to be aware of pituitary involvement in genuine COVID-19 infections. The two cases of Aliberti et al. (1) and Piñar-Gutiérrez et al. (4) should prompt greater caution when vaccinating patients with pituitary adenomas because of the additional risk of apoplexy that could be caused by the vaccine (5). Currently, this remains true as the need for COVID vaccination persists, at least seasonally, in the years to come. Other cases could emerge in the future and may better explain the interrelation that might exist between pituitary ACE-2 receptors, the coronavirus, and the anti-COVID-19 vaccine.

## Author contributions

AT drafted the manuscript. All authors contributed to the article and approved the submitted version.
